# Supplementary material for: Multi‐dataset identification of innovative feature genes and molecular mechanisms in keratoconus
Source: J Cell Mol Med. 2024 Sep 19;28(18):e70079. doi: 10.1111/jcmm.70079 (PMC11412914; doi:10.1111/jcmm.70079)
Supplement: Supplementary file 2 — Material S2. [file JCMM-28-e70079-s002.zip › 7.GSEA analysis/6.SNORA74B/KEGG_ACUTE_MYELOID_LEUKEMIA.html]

Details for gene set KEGG\_ACUTE\_MYELOID\_LEUKEMIA[GSEA]

|  || Dataset | SNORA74B.group.cls#h\_versus\_l |
| Phenotype | group.cls#h\_versus\_l |
| Upregulated in class | l |
| GeneSet | KEGG\_ACUTE\_MYELOID\_LEUKEMIA |
| Enrichment Score (ES) | -0.36011532 |
| Normalized Enrichment Score (NES) | -1.4705693 |
| Nominal p-value | 0.06916996 |
| FDR q-value | 0.31469175 |
| FWER p-Value | 0.824 |
Table: GSEA Results Summary

  

Fig 1: Enrichment plot: KEGG\_ACUTE\_MYELOID\_LEUKEMIA      
 Profile of the Running ES Score & Positions of GeneSet Members on the Rank Ordered List

  

| SYMBOL | TITLE | RANK IN GENE LIST | RANK METRIC SCORE | RUNNING ES | CORE ENRICHMENT || 1 | PIK3R5 | na | 170 | 0.800 | 0.0936 | No |
| 2 | FLT3 | na | 4865 | 0.230 | -0.0135 | No |
| 3 | SOS1 | na | 5971 | 0.193 | -0.0216 | No |
| 4 | BRAF | na | 8089 | 0.134 | -0.0662 | No |
| 5 | PIK3R1 | na | 8116 | 0.133 | -0.0506 | No |
| 6 | CCNA1 | na | 8695 | 0.120 | -0.0525 | No |
| 7 | SPI1 | na | 8726 | 0.120 | -0.0386 | No |
| 8 | IKBKB | na | 8763 | 0.119 | -0.0250 | No |
| 9 | MTOR | na | 8938 | 0.115 | -0.0159 | No |
| 10 | AKT2 | na | 9380 | 0.105 | -0.0157 | No |
| 11 | CEBPA | na | 9683 | 0.099 | -0.0122 | No |
| 12 | KRAS | na | 9766 | 0.097 | -0.0026 | No |
| 13 | TCF7L2 | na | 12663 | 0.051 | -0.0798 | No |
| 14 | RPS6KB2 | na | 12925 | 0.048 | -0.0815 | No |
| 15 | PIK3R2 | na | 12946 | 0.047 | -0.0762 | No |
| 16 | PIK3CA | na | 13079 | 0.046 | -0.0743 | No |
| 17 | RPS6KB1 | na | 13499 | 0.042 | -0.0813 | No |
| 18 | SOS2 | na | 13685 | 0.039 | -0.0818 | No |
| 19 | MAP2K2 | na | 14904 | 0.025 | -0.1138 | No |
| 20 | STAT5B | na | 15213 | 0.022 | -0.1199 | No |
| 21 | CCND1 | na | 15223 | 0.022 | -0.1174 | No |
| 22 | PIK3CB | na | 15769 | 0.017 | -0.1311 | No |
| 23 | CHUK | na | 16402 | 0.010 | -0.1480 | No |
| 24 | MAPK3 | na | 16753 | 0.007 | -0.1572 | No |
| 25 | PPARD | na | 17698 | -0.003 | -0.1842 | No |
| 26 | JUP | na | 17968 | -0.006 | -0.1912 | No |
| 27 | AKT1 | na | 18366 | -0.010 | -0.2014 | No |
| 28 | PIK3CG | na | 18884 | -0.016 | -0.2144 | No |
| 29 | BAD | na | 19265 | -0.021 | -0.2228 | No |
| 30 | HRAS | na | 19699 | -0.025 | -0.2322 | No |
| 31 | RARA | na | 20023 | -0.029 | -0.2380 | No |
| 32 | PIK3CD | na | 20354 | -0.032 | -0.2435 | No |
| 33 | MAPK1 | na | 20606 | -0.035 | -0.2464 | No |
| 34 | IKBKG | na | 20820 | -0.038 | -0.2479 | No |
| 35 | RAF1 | na | 21074 | -0.041 | -0.2502 | No |
| 36 | PML | na | 23015 | -0.066 | -0.2981 | No |
| 37 | KIT | na | 23499 | -0.073 | -0.3030 | No |
| 38 | GRB2 | na | 24997 | -0.098 | -0.3341 | No |
| 39 | MYC | na | 25534 | -0.108 | -0.3363 | No |
| 40 | STAT3 | na | 26362 | -0.124 | -0.3448 | Yes |
| 41 | NFKB1 | na | 26534 | -0.128 | -0.3339 | Yes |
| 42 | TCF7L1 | na | 27185 | -0.144 | -0.3350 | Yes |
| 43 | PIM1 | na | 27699 | -0.157 | -0.3305 | Yes |
| 44 | ARAF | na | 27916 | -0.162 | -0.3167 | Yes |
| 45 | NRAS | na | 27969 | -0.164 | -0.2981 | Yes |
| 46 | STAT5A | na | 28673 | -0.185 | -0.2956 | Yes |
| 47 | TCF7 | na | 28810 | -0.189 | -0.2762 | Yes |
| 48 | EIF4EBP1 | na | 29055 | -0.196 | -0.2591 | Yes |
| 49 | PIK3R3 | na | 29453 | -0.208 | -0.2449 | Yes |
| 50 | RELA | na | 29651 | -0.214 | -0.2243 | Yes |
| 51 | PIM2 | na | 30474 | -0.244 | -0.2179 | Yes |
| 52 | RUNX1 | na | 31103 | -0.273 | -0.2024 | Yes |
| 53 | MAP2K1 | na | 31129 | -0.275 | -0.1693 | Yes |
| 54 | RUNX1T1 | na | 32078 | -0.327 | -0.1564 | Yes |
| 55 | ZBTB16 | na | 32824 | -0.378 | -0.1314 | Yes |
| 56 | LEF1 | na | 34351 | -0.633 | -0.0974 | Yes |
| 57 | AKT3 | na | 34657 | -0.880 | 0.0020 | Yes |
Table: GSEA details [plain text format]

  

Fig 2: KEGG\_ACUTE\_MYELOID\_LEUKEMIA      
 Blue-Pink O' Gram in the Space of the Analyzed GeneSet

  

Fig 3: KEGG\_ACUTE\_MYELOID\_LEUKEMIA: Random ES distribution      
 Gene set null distribution of ES for **KEGG\_ACUTE\_MYELOID\_LEUKEMIA**
